# Are Myxobacteria intelligent?

**DOI:** 10.3389/fmicb.2013.00335

**Published:** 2013-11-12

**Authors:** Dale Kaiser

**Affiliations:** ^1^Department of Biochemistry, Stanford University School of MedicineStanford, CA, USA; ^2^Department of Developmental Biology, Stanford University School of MedicineStanford, CA, USA

**Keywords:** bacterial swarm, cell Polarity, gliding motility, reversal of direction, timer for reversals, rafts of cells, multicellular mounds

## Abstract

“Intelligence” is understood in different ways. Because humans are proud of their ability to speak, intelligence often includes the ability to communicate with others, to plan for the future, and to solve frequently encountered problems. Myxobacteria are among the most socially adept and ubiquitous of bacteria that live in the soil. To survive in nature, Myxobacteria communicate with their peers, using signals that elicit specific responses. Both swarming-growth and starvation-induced fruiting body development depend upon the specificity and effectiveness of signals passed between cells. Dynamic swarms spread outward, forming regular multi-cellular and multi-layered structures as they spread. Several different extra-cellular signals have been identified for fruiting body development and one is hypothesized for swarm development. Some extra-cellular signals are small, diffusible molecules. Others are protein molecules. The swarm signal appears to consist of structurally complex, protein to protein, contact junctions between pairs of side by side aligned cells. Each junction persists for less than a minute before disconnecting. After separating, both cells move on to make similar, transient connections with other cells. Eventually, the signal spreads across a prescribed population of communicating cells.

## INTRODUCTION

**FIGURE 1:**
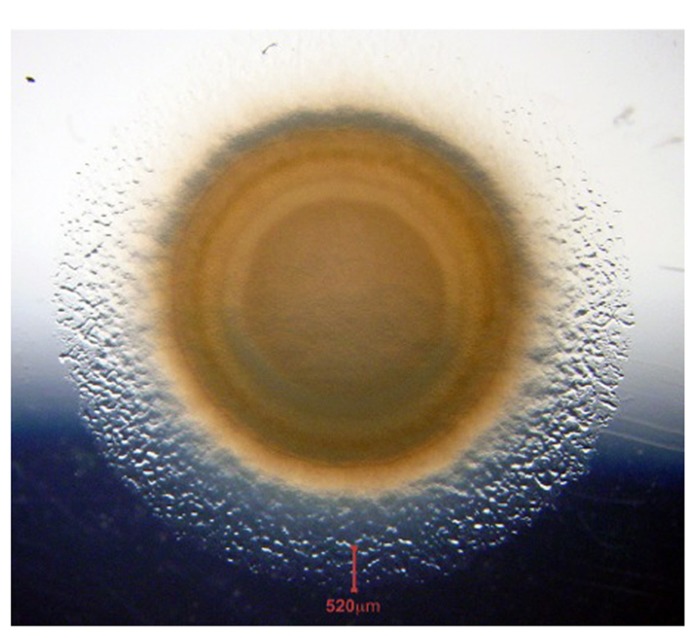
**Swarm of wild type *M. xanthus* on a CTT agar plate. The photo was taken after 7 days of incubation at 20°C.** The vertical red line at the bottom of the panel marks the 520 μm wide edge of the annulus. We observe annular cells to be growing exponentially at their maximum rate, as each cell continues to move. The swarm is a dynamic collection of interacting cells.

Myxobacteria are facultative multicellular organisms, a quality particularly useful for the study of signaling between cells. Given a complete medium for liquid culture, these delta-proteobacteria (Goldman et al., 2006) grow as independent rod-shaped cells; growing cells are 5–7 μm in length and 0.5 μm in diameter. On a solid surface, these elongated cells move cooperatively in a multicellular swarm, while individual cells prefer to move in the direction of their long axis. Once they have grown to moderate cell densities, adjacent swarm cells tend to align with each other and to form rafts of moving cells (Kaiser and Warrick, 2011). Non-motile mutants form dense, sharp-edged, ordinary colonies whose cells are heaped on each other, lack organization (no rafts), and do not swarm. Moreover, non-motile mutants are unable to signal each other or to form organized fruiting body-like structures (Kroos et al., 1988). Thus, individual swarm cells seem to be recognizing and specifically interacting with each other in a swarm and as they proceed to develop fruiting bodies (Hagen et al., 1978; LaRossa et al., 1983). A swarm can spread at the same rate for more than 300 h (Kaiser and Warrick, 2011). The average speed of individual cells in a swarm can be measured accurately by the steady-state rate of swarm expansion. **Figure 1** illustrates the perfect radial symmetry of an expanding swarm of *Myxococcus xanthus*.

Among the sequenced delta-proteobacterial genomes, only the myxobacteria have the capacity to develop multicellular fruiting bodies as well as the capacity to differentiate spores. How can anyone apply the concept of intelligence to the behavior of Myxobacteria, or to any other microbe for that matter, when “intelligence” is clearly understood only for humans and the higher vertebrates that have large brains? To deal with that question, H. S. Jennings (Jennings, 1976) took a completely experimental approach. He set aside the philosophically daunting problems of conscious self awareness by asking, “What can we learn by observing the motility of single cells directly?” Jenning’s investigative approach has proven scientifically fruitful; it has led science into an era of molecular motors, of molecular genetics, and of molecular cell biology. Applied to communication between cells, which is to the transfer of information between two cells (a sender and a receiver), Jenning’s approach has the advantage of facilitating the construction of relatively simple but rigorous bioassays. This is an assay in which pure compounds are added to complex receiver cells, and their response observed. The assay performs well even though the bio-molecular mechanism underlying the cell’s response to a pure compound is unknown and probably will remain unknown for some time to come. Intelligence can then be understood in the sense of having the ability to acquire and to apply knowledge – in this case knowledge of the crude physical–biochemical state of the cell. As powerful as the method may be, I believe that rigor stops at a physical-biochemical understanding of large, single molecules. The action of systems of enzymes or systems of structural proteins, of metabolic chains, of chromatin, or of metabolic diseases is limited to investigation by modeling based on a small number of parameters. As a principle of mathematical logic, no model can be demonstrated by any conceivable set of experiments. However, as plausible models, they can be supported more and more strongly by experimental data. Progress is possible whenever a more plausible model can be used and its testing can be resumed.

Returning to the myxobacteria as models of multicellular development, each of the 45 different species of myxobacteria, which differ strikingly from each other, build or self- organize their own fruiting bodies. For more than 200 years, the species have been reliably distinguished simply by the morphology of their fruiting bodies. Recent 16S RNA phylogenies have improved the accuracy of the morphological species descriptions (Shimkets and Woese, 1992). In principle, the different species offer biologically independent tests of the same signals. Proceeding forward through the several stages of *M. xanthus *fruiting body development, when cell growth begins to outrun it’s food supply, swarm cells change their behavior in major ways: (1) the swarm stops expanding outward and instead retreats, (2) it migrates inward, and (3) it builds hundreds of fruiting bodies, each with hundreds of thousands of spores. Moreover, a swarm that has begun to develop appears to allocate the precious resources that remain to it. They appear to allocate enough of each nucleotide triphosphate to DNA synthesis in order that each spore, ultimately, will contain two complete copies of the chromosome (Tzeng and Singer, 2005). And because more than 30 new proteins are made during fruiting body development, some ribo- and some deoxy-ribonucleotide triphosphates are also set aside for developmental protein synthesis. It is as if sensing a deficiency of any amino-acylated tRNA, the swarm initiates the developmental program for fruiting bodies. *M. xanthus *uses its stringent response (Singer and Kaiser, 1995) to initiate a cascade of enhancer- binding proteins, or EBPs (Giglio et al., 2011). The cascade organizes the transitions from exponential growth to pre-aggregation, to mound building and finally to sporulation within the mounds. EBPs are specific transcriptional activators that work with sigma-54-RNA polymerase to activate transcription at designated sigma-54 promoters. In response to an activating signal, such as phosphorylation by a histidine kinase sensor protein, EBPs use the energy of ATP hydrolysis to form a transcription-competent open promoter complex. Cascade EBPs auto-regulate: first, the expression of a downstream EBP is activated at the proper time by a preceding EBP in the cascade and, second, three of the EBPs positively regulate their own expression. Early detection of approaching starvation seems to limit spore formation because it is generally observed that no more than 0.1–1% of the cells initiating fruiting body development differentiate into spores. Therefore, it is suggested that several of the cascade’s sensor kinases measure the level of intermediary metabolites that are indicative of starvation’s approach and signal the need to make fruiting bodies. These signals are still hypothetical and they await construction of specific bioassays.

It is believed that later when the soil becomes more conducive to the growth of prey bacteria, the myxospores germinate, the growing cells feed on the new prey, and assemble a new swarm. We find that most of the signaling is found within organized groups of cells in the multicellular rafts and multilayered mounds of a steady-state swarm. The cascade of EBPs may be giving the most appropriate response to the swarm signal.

## MATERIALS AND METHODS

### BACTERIA

Cultures of *M. xanthus*, DK1622, and several of its mutants were grown as described in Kaiser and Warrick (2011). Bacteria were propagated as swarms by inoculating an agar plate with bacteria harvested from the edge of a mature swarm using the tip of a sterilized round toothpick, and incubating at 20°C. Most likely, clusters of aligned cells carried on the toothpick helped nucleate the swarm. Time-lapse photo-microscopy was carried out as described in Kaiser and Warrick (2011).

### CONSTRUCTING A BIOASSAY

In 1978, we set out to find which extracellular signals *M. xanthus *employs, using a simple bioassay. Two signals were identified from a set of conditionally defective mutants unable to form spore-filled fruiting bodies in single-species pure culture (Hagen et al., 1978; LaRossa et al., 1983). When different mutants were mixed with wild-type cells or with other mutants, some of those mixtures were able to form fruiting bodies with spores. Pairwise mixing of 57 mutants identified two extracellular signals: A and C.

## RESULTS

### A-SIGNAL

Medium conditioned by *Myxococcus* development was found to include both a heat-stable and a heat-labile form of A-signal activity. In 1992, Plamann et al. found that heat-labile A-signal was a mixture of proteases and proteins that were degraded by the proteases (Plamann et al., 1992). That same year Kuspa et al. showed that heat-stable A-signal was a set of six amino acids and small peptides containing those amino acids (Kuspa et al., 1992). It appears that amino acids are the primary A-signal molecules, while the extracellular release of proteases and proteins generates first peptides, then A-signal amino acids.

Singer and Kaiser (1995) found that fruiting body development is induced by starvation and that A-signal helps *M. xanthus *assess the nutrient available for developmental protein synthesis. When *Myxococcus* is challenged by starvation, it must choose between initiating fruiting body development with differentiation of spores or slow growth at a rate compatible with the level of nutrient available. In nature, myxobacteria feed on particulate organic matter in the soil. Because death follows for the great majority of cells (more than 99%), the option of choice depends on each cell’s projection of future nutrient availability. If nutrient is on its way to exhaustion, then slowing growth to match the level of residual nutrient leads to slower and slower growth, until death from starvation follows. Manoil found that limitation for any amino acid or starvation for carbon, energy, or phosphorous induces fruiting body development (Manoil and Kaiser, 1980). Neither O2 deprivation nor purine or pyrimidine starvation induces development (Kimsey and Kaiser, 1991). Since a complete set of amino-acylated tRNAs is needed for protein synthesis, the absence of one or more amino-acylated tRNA is readily perceived. In *M. xanthus*, as in many other bacteria, the absence or shortage of any one of the charged tRNAs being called for by messenger RNA causes a ribosome to synthesize guanosine tetra- (and penta-) phosphate, (p)ppGpp. Pyrophosphate, or P~P, is transferred from ATP to GTP, and a stringent response is triggered. Singer showed that (p)ppGpp was both necessary and sufficient to initiate fruiting body development (Singer and Kaiser, 1995). Giglio et al. found that development is initiated and propagated by a sequence of enhancer binding proteins, responding to the level of A-signal (Giglio et al., 2011). Recently, Sarwar confirmed that *M. xanthus *uses a stringent response and the cell-density-dependent A-signal to predict the future nutrient availability (Sarwar and Garza, 2012).

A-signal is water soluble and diffusible, and the model emerging from Giglio et al. is that some threshold number of starving cells is needed to begin development (Giglio et al., 2011). A census of the cell population is then taken to determine whether the consensus is adequate. By sensing the level of nutrient available, each cell naively casts a vote as to whether it is time to begin fruiting body development. When it produces A- signal, it casts its vote for development. But the vote is advisory only, for if nutrient is added, all the cells begin to grow again; they haven’t yet committed themselves to sporulation. By definition, quorum signals are soluble and diffusible and they are used by many bacteria to determine the number of bacteria in their neighborhood belonging to their species as well as the number that belong to different species. *Bacillus subtilis*, for example, uses a particular pentapeptide, PhrC, to regulate competence for DNA transformation and to regulate the initiation of sporulation (Auchtung and Grossman, 2008), with each signal dependent upon a particular biochemical model. *Vibrio harveyi *and *Vibrio cholerae *use a variety of homoserine lactones as quorum sensors (Hammer and Bassler, 2008). Two questions arise for every quorum sensor: (1) What is the chemical identity of the signal molecule and (2) what is the biochemical model for the receiver?

### C-SIGNAL

The C-signal, rather than sensing a quorum, is a morphogenetic paracrine signal. C-signal is required for cellular aggregation, spore differentiation, and gene expression induced by starvation (Kim and Kaiser, 1990b,d). Surprisingly, cell motility (apparently A-motility) is required for proper intercellular transmission of the signal (Kim and Kaiser, 1990c). Due to the complexity of its action and the many poorly understood proteins necessary for *M. xanthus *development, only fragments of the C-signal transduction pathway can be written down; a number of fragments are compiled in one review (Søgaard-Andersen, 2008). C-signal, itself, is cell-bound, and signal exchange requires direct contact between two cells, unlike the A-signal that can diffuse between cells. Active C-signal was purified from the membrane fraction of whole cells, using a detergent to dissolve the signal protein. The model bioassay depended upon restoring aggregation and sporulation to a mutant lacking a *csgA *gene (Kim and Kaiser, 1990c). The mutants arrested fruiting body development before aggregation (Kaiser, 2003) and they formed very few, if any, spores. A 17 kDa protein was purified from starved wild type cells that could restore activity to *csgA *mutants (Kim and Kaiser, 1990c). C-signal activity was not recovered from extracts of growing cells (not starved) or from *csgA *mutants (Kim and Kaiser, 1990a). Subsequent experiments showed that p17 is the molecular signal and that it is produced by the PopC cell-surface protease acting on p25 (Kruse et al., 2001). C-signal carries information as to cell density and to cell position with respect to other cells. It was a surprise to find that non-motile mutants of *M. xanthus *arrested fruiting body development at exactly the same morphological stage as the *csg*A mutants (Kroos et al., 1988). This observation suggested that C-signaling might occur only between aligned cells in end-to-end contact. Seung Kim tested this hypothesis by mechanically placing non-motile cells into end-to- end alignment (Kim and Kaiser, 1990b). The asymmetry of the long rod-shaped *M. xanthus *cells was used to orient them lengthwise as they tumbled from suspension into the narrow grooves produced by scoring agar with a fine grained aluminum oxide abrasive paper. Phase contrast microscopy revealed that cells, which had settled into the grooves, were indeed oriented with their long axes parallel to the axis of the groove (Kim and Kaiser, 1990b). Because the grooves were 5–10 μm wide, cells were also found lying side-by-side in the grooves. For that reason, the experiment did not exclude C-signaling between the sides of two cells. Further experiments are required to test whether side-by-side contacts are involved in C-signaling. A related difficulty is that a C-signal receptor in the receiving cell has yet to be identified. Gronewold discovered positive feedback in the C- signal reception circuit, controlled by the *act *operon of 5 co-transcribed genes (Gronewold and Kaiser, 2001, 2002). Positive feedback appears to raise progressively the number of C-signal molecules per cell from a few at 3 h post starvation to several hundred by 18 h (Gronewold and Kaiser, 2001, 2002). This rise coordinates C-signal-dependent gene expression, and eventually it triggers spore differentiation with a concomitant loss of cell motility (Kroos and Kaiser, 1987; Julien et al., 2000). There may be a functional similarity between C- signal, the PatS- and the HetN-dependent formation of heterocysts in the filamentous, nitrogen-fixing cyanobacteria Anabaena PCC 7120 (Aldea et al., 2008)

### MIGHT FOCAL ADHESIONS TRANSMIT A SIGNAL?

In 1977, Jonathan Hodgkin, then a postdoctoral fellow in my laboratory, discovered that CglB, a protein that is essential for A-motility, and later found to be an outer membrane lipoprotein, could be transferred from one cell to another cell, provided the cells had come into end-to-end contact with each other (Hodgkin and Kaiser, 1977). However, only the CglB^+^ phenotype was transferred, which was detected by a gain of A-motility; the *cglB *gene was not transferred! Hodgkin found that the CglC, CglD, CglE, and CglF phenotypes were capable of being similarly transferred (Hodgkin and Kaiser, 1977). Hodgkin also found that the Tgl protein and not the *tgl *gene is transferred (Hodgkin and Kaiser, 1979). Tgl protein was shown to be an assembly factor that non-covalently linked 12–14 PilQ monomers together into an active PilQ multimer secretin (Rodriguez-Soto and Kaiser, 1997a,b). Subsequently, it was shown that substantial amounts of Tgl and CglB proteins were being transferred as a result of the transient contact with a mutant recipient – enough of each to restore full A^+^S^+^ motility (Nudleman et al., 2005). Recently, two host proteins, TraA and TraB, were shown to be essential in both donor and recipient for contact-mediated, outer-membrane lipoprotein transfer of this type (Pathak et al., 2012). In addition, TraA and TraB were found to mediate the transfer of lipids that modify swarming (Pathak et al., 2012). Although CglB, CglC, and CglD proteins have type II signal sequences, the transfer of CglE and CglF proteins, which have type I signal sequences, suggests that lipid molecules in addition to proteins without any lipid modification, like Tgl, can be transferred from one cell to another, provided they have been targeted to the outer-membrane (Pathak and Wall, 2012). Bhat et al.have identified many beta-barrel and lipoproteins in the outer membrane of *M. xanthus *by LC- MS/MS (Bhat et al., 2011). Because only a small fraction of *M. xanthus *outer membrane proteins can be transferred by cell contact, one can imagine that the few that can be transferred are not only exposed to other cells but organized for signaling to them.

**FIGURE 2:**
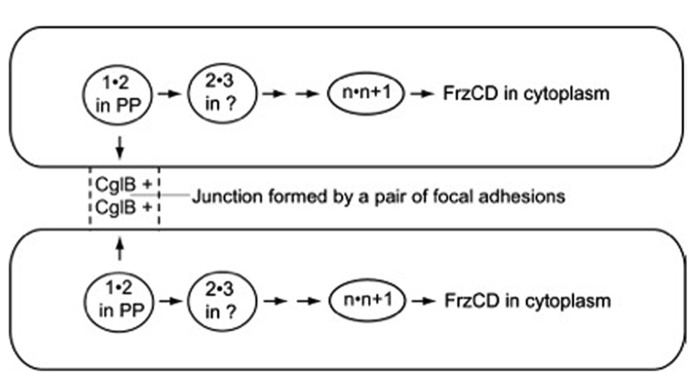
**The path followed by the signal postulated to synchronize the pacemakers of the cell pair shown.** Arrows point toward the next pair of numbered A-motility proteins to bind together. Adhesion proteins are represented in the figure by numbers that indicate their position in the sequence of pairwise binding steps, unless their location is established, like CglB and FrzCD. 1"2 is the first pair of proteins to bind, 2"3 is the second pair, *n"n* + 1 is the next to last pair, and *n* + 1"FrzCD is the last pair. FrzCD is a methylated, regulatory protein, not an A-motility protein. The two cells shown are joined for a short time, just long enough to complete the whole series of binding steps that run from CglB to FrzCD, and through all the membrane bound compartments of the cell. PP, in the diagram, represents the cell’s periplasmic compartment.

In the fore-mentioned group of exposed and organized proteins, CglB, CglC, CglD, CglE, and CglF are proposed to link a pair of adjacent cells together in a particular way – to link them through a pair of multi-protein structures known as focal adhesions (Mignot et al., 2005, 2007) and to link them, thus, for less than a minute before the two cells separate and move on to link similarly to other cells, and signal to them. The focal adhesions are found on the sides of *M. xanthus *cells; each adhesion can be seen as a discrete series of fluorescent foci. Despite the multitude of different proteins that cluster in the focal adhesions, no amino acid sequences related to microtubule-based kinesin motors or actin-based myosin motors (Vale and Milligan, 2000) have been identified (see Luciano et al., 2011, for example). Indeed, the 15 or more A-motility proteins in the clusters associated with focal adhesions seem well-suited for a signaling pathway for two reasons. One, many of the proteins can bind one another in specific pairs as measured by GST affinity chromatography, identified in Table 1 of Nan et al. (2010). Two, different binding proteins are found to favor localization in different subcellular compartments of an *M. xanthus *cell. Proceeding inward, they are found on the outer surface of the outer membrane, in the periplasmic space, on the outer surface of the peptidoglycan sacculus, associated with the inner membrane, and finally in the cytoplasm, where the pacemaker proteins are located (Kaiser and Warrick, 2011). Due to their broad spatial distribution, the hypothetical signal would be capable of linking methylated-FrzCD in the pacemaker of one cell through pairs of protein 1 transiently bound to protein 2 links to CglB – one of the 15 A-motility proteins found in a focal adhesion – on the surface of that cell. CglB etc. on the surface of the first cell could assemble together with CglB etc. on the surface of the second cell, transiently forming a junction with a specific structure between the 2 cells. From that junction, the signal would link through the same series of protein 1" protein 2 pairs until it reached FrzCD in the pacemaker of the second cell. When completed, as shown in **Figure 2**, the series of signal links could plausibly bring the pacemakers of both cells to the same phase of their oscillatory cycles. The circuit of **Figure 2** offers a concrete and thus testable example of the signaling alternative.

## CONCLUDING REMARKS

How intelligent are myxobacteria? In sum, the A (quorum sensing) and the C (multicellular structure building) signal molecules have been chemically identified. Models of their signaling pathways, based on experimental data, are offered. A new swarm signal is proposed that is based on the structure of focal adhesions and the observed movement patterns of swarm cells. All the A-motility proteins on the swarm signaling pathway have been identified along with their ability to bind the next protein on the pathway, as well as each of their locations within sub-cellular compartments used to assign their position in the sequential order of protein-protein binding steps. Experiments are in progress to test the relevance of the proposed signal to the observed dynamics of the swarm. Together, these three signaling pathways regulate the behavior of individual cells so that each cell can contribute to the social order of the swarm or of the fruiting body to ensure their survival. Clearly, myxobacteria have evolved attributes that can be considered signs of intelligence.

## Conflict of Interest Statement

The author declares that the research was conducted in the absence of any commercial or financial relationships that could be construed as a potential conflict of interest.
